# Correction: Addressing the ethical issues raised by synthetic human entities with embryo-like features

**DOI:** 10.7554/eLife.27642

**Published:** 2017-04-13

**Authors:** John Aach, Jeantine Lunshof, Eswar Iyer, George M Church

Aach J, Lunshof J, Iyer E, Church GM. 2017. Addressing the ethical issues raised by synthetic human entities with embryo-like features. *eLife* **6**:e20674. doi: 10.7554/eLife.20674.

Published 21, March 2017

Three changes have been made to correct inaccuracies in citations of particular scientific research papers or commentaries. Five additional changes have been made to correct references to particular sections, rules, and page numbers of prior ethics commission reports.

Changes made to correct citations of particular research papers or commentaries

*Change 1:* The term and concept of "gastruloid," which we referred to (Pera et. al., 2015), was actually first introduced in (van den Brink et.al., 2014) to describe systems developed in the laboratory of Martinez Arias of small aggregates of mouse embryonic stem cells that appear to recapitulate early embryonic events.

The sentence in the text that introduced this term has been changed to begin:

The entities generated by Warmflash et al. (called “gastruloids” by Pera et al., a term used earlier by van den Brink et.al., 2014) exhibit a PS …

The original beginning of this sentence is shown for reference:

The entities generated by Warmflash et al. (called “gastruloids” in Pera et al., 2015) exhibit a PS …

In connection with *Change 1* above, the following citation has been added to the Reference list:

van den Brink SC, Baillie-Johnson P, Balayo T, Hadjantonakis AK, Nowotschin S, Turner DA, Martinez Arias A. 2014. Symmetry breaking, germ layer specification and axial organisation in aggregates of mouse embryonic stem cells. Development **141**: 4231-4242. doi: http://dx.doi.org/10.1242/dev.113001

*Changes 2 and 3:* Two sentences citing the paper (Denker, 2014) have been changed to more accurately reflect the views expressed in that paper. One sentence has been changed to avoid an incorrect suggestion that (Denker, 2014) calls for re-evaluation of the 14-day rule. In the other, the citation of (Denker, 2014) has been removed because that paper anticipates that human pluripotent cells could exhibit totipotency.

For change 2, a sentence in Discussion now begins:

While the ethical issues raised by SHEEFs have called attention to the 14-day rule (Denker, 2014; Hyun et al., 2016; Pera et al., 2015), …

The original beginning of this sentence is shown for reference:

While the ethical issues raised by SHEEFs have led to calls for re-evaluation of the 14-day rule (Denker, 2014; Hyun et al., 2016; Pera et al., 2015), …

For change 3, a sentence in the introduction now begins:

Commentaries (Hyun et al., 2016; Pera et al., 2015) have noted that these experiments represent an important advance …

The original beginning of this sentence is shown for reference:

Commentaries (Denker, 2014; Hyun et al., 2016; Pera et al., 2015) have noted that these experiments represent an important advance …

Changes made to correct references to particular sections, rules, or page numbers in prior ethics commission reports

*Change 4:* A citation in a sentence that describes the significance attributed to the primitive streak in the Warnock report has been changed to:

(Warnock, 1984, sections 11.19-11.22;National Institutes of Health, 1994, chap. 3, pp. 46-8).

The original citation is shown for reference:

(Warnock, 1984, section 11.19;National Institutes of Health, 1994, chap. 3, pp. 46-8).

*Change 5:* A citation of a rule regarding parthenogenesis and androgenesis in the National Academy of Science’s 2005 guidelines has been changed to:

(Warnock, 1984, Section 12.10, p. 72; National Institutes of Health, 1994, p. xv; National Research Council, 2005, Section 4.5).

The original citation is shown for reference:

(Warnock, 1984, Section 12.10, p. 72; National Institutes of Health, 1994, p. xv; National Research Council, 2005, Section 4.5, p. 23).

*Change 6:* The text of a sentence citing rule numbers for a particular instance of the 14-day rule in the National Academy of Science’s guidelines of 2005, 2008, and 2010 has been changed to:

… two separate statements of the 14-day rule, one in connection with embryo culturing (rule 1.2(c) in 2005; 1.3(c) in 2008 and 2010),

The original sentence text is shown for reference:

… two separate statements of the 14-day rule, one in connection with embryo culturing (rule 1.2(c) in 2005 and 2008; 1.3(c) in 2010),

*Change 7:* The page number of a sentence of the National Institutes of Health’s 1994 report that described opinions regarding the moral significance of the onset of heartbeat has been changed to:

(National Institutes of Health, 1994, chap. 3, p.38 and p. 47)

The original citation is shown for reference:

(National Institutes of Health, 1994, chap. 3, p.38 and pp. 46-7)

*Change 8:* Page numbers that refer to sections of an appendix to the 1979 report of the Ethics Advisory Board that discuss cloning have been changed to:

(Walters, 1979, pp. 44ff, 51-52; Warnock, 1984, section 12.14, p.73)

The original citation is shown for reference:

(Walters, 1979, pp. 44ff, 50; Warnock, 1984, section 12.14, p.73)

